# AKT1 Gene Polymorphisms and Obstetric Complications in the Patients with Schizophrenia

**DOI:** 10.4306/pi.2009.6.2.102

**Published:** 2009-06-30

**Authors:** Eun-Jeong Joo, Kyu-Young Lee, Seong-Hoon Jeong, Myoung-Sun Roh, Se Hyun Kim, Yong-Min Ahn, Yong Sik Kim

**Affiliations:** 1 Department of Psychiatry, Eulji University School of Medicine, Daegeon, Korea.; 2 Department of Psychiatry, Dongguk University College of Medicine, Goyang, Korea.; 3 Department of Psychiatry and Behavioral Science and Institute of Human Behavioral Medicine, Seoul National University College of Medicine, Seoul, Korea.

**Keywords:** AKT1 gene, Association, Lewis scale, Obstetric complication, Schizophrenia

## Abstract

**Objective:**

We performed a genetic association study with schizophrenic patients to investigate whether the V-akt murine thymoma viral oncogene homolog 1 (AKT1) gene plays a role in obstetric complications.

**Methods:**

One-hundred-eighty patients with schizophrenia (male, 113; female, 67) were included. All patients fulfilled DSM-IV criteria for schizophrenia. Obstetric complications were measured by the Lewis scale. Prenatal and perinatal information was retrospectively collected from the patients' mothers. We selected six single nucleotide polymorphisms (SNPs) for the AKT1 gene: SNP1 (rs3803300), SNP2 (rs1130214), SNP3 (rs3730358), SNP4 (rs 1130233), SNP5 (rs2494732), and SNPA (rs2498804). The genotype data were analyzed for an association with the Lewis total score in terms of allele, genotype, and haplotype distribution.

**Results:**

The mean total Lewis scores were 1.30±1.61 for males and 1.54±1.87 for females. Higher total score tended to be correlated with an earlier age of onset of schizophrenia in females. In the total sample, no SNP was associated with obstetric complications. However, the additional analyses for male and female subgroups found a significant association between SNPA and SNP4 and Lewis score in females (p=0.02 for SNPA, p=0.04 for SNP4). The SNP5-SNPA haplotype showed a positive association with obstetric complications (p=0.03) in the female patient group.

**Conclusion:**

We found an association between SNPs in the AKT1 gene and total Lewis score measuring obstetric complications in female patients with schizophrenia. Because these findings did not survive a correction for multiple testing, the significance should be interpreted carefully and replication studies are required.

## Introduction

The prevalence of obstetric complications is higher in patients with schizophrenia than in the general population.^1^,^2^ A nongenetic developmental understanding of schizophrenia has usually considered prenatal, perinatal, and postnatal obstetric complications,^3^ but serious obstetric complications interact with genetic factors to increase the risk for schizophrenia.^1^,^4^ Various kinds of obstetric complications occur, but most cause hypoxia to the subject, and hypoxia is an important external factor influencing schizophrenia susceptibility.^5^ Molecular systems regulating hypoxia are especially crucial for development of the brain during the intrauterine period; therefore, dysfunction of this system could be one of the causes of schizophrenia.^6^ About 50% of candidate genes for schizophrenia also appear to be associated with ischemia-hypoxia regulation and vascular expression.^5^

The development of schizophrenia involves an interaction of genetic and environmental factors, and obstetric complications may interact as an environmental factor with the genes related to schizophrenia. In addition, obstetric complications may be a phenotypic result of genes related to intrauterine fetal development and perinatal events. A recent study by Nicodmus et al.^7^ found an interaction between hypoxia-regulated vascular-expression genes and serious obstetric complications in schizophrenia risk. They found significant evidence for a gene x environment interaction in V-akt murine thymoma viral oncogene homolog 1 (AKT1), brain-derived neurotrophic factor (BDNF), dystrobrevin binding protein 1 (DTNBP1), and glutamate receptor metabotrophic (GRM) genes in developing schizophrenia.

The AKT1 gene is located on chromosome 14q32.32 and is known to mediate growth factor-induced neuronal survival in the developing nervous system. The activation of AKT1 occurs through phosphatidylinositol 3-kinase (PI3K). The PI3K/AKT pathway plays a crucial role in signal transduction of cell growth, survival, and metabolism.^8^ The functional role of the PI3K/AKT pathway in ischemia-hypoxia regulation and neuroprotection has been studied.^9^-^13^ AKT blocks apoptotic stimuli by inactivating pro-apoptotic proteins. AKT also exerts antiapoptotic effects by activating endothelial nitric oxide synthase (eNOS).^14^

Previous studies suggested that AKT1 plays an important role in the pathophysiology of schizophrenia, and AKT phosphorylation in the rat frontal cortex after acute and chronic treatment with MK-801 are well known animal models of schizophrenia.^15^ Reduced AKT protein and RNA levels have been reported in studies of postmortem brains of patients with schizophrenia.^16^,^17^ Kalkman suggested that reduced activity of the PI3K/AKT pathway makes the brain more susceptible to viral infections, anoxia, and obstetric complications.^18^ The AKT1 gene has also been reported as a susceptibility gene for schizophrenia in genetic association studies.^19^-^21^

We previously collected Lewis scale data for patients with schizophrenia. These patients have been genotyped for six SNPs of AKT1 to identify a possible association with a schizophrenia diagnosis. In this paper, we analyzed these data to identify the association between AKT1 gene function and obstetric complications.

## Methods

### Subjects

The patients with schizophrenia were collected from three large psychiatric clinics in Korea. The same research nurse collected clinical information including medical records and additional information from the psychiatrist in charge of treatment of the patient. This information was reviewed in order to get consensus diagnoses by two psychiatrists. Not all but most subjects were actually interviewed using DIGS-K (Diagnostic Interview for Genetic Studies-Korean version).^22^ This information also was used for consensus diagnosis. All patients fulfilled the DSM-IV diagnostic criteria for schizophrenia, and both the diagnosis of schizophrenia and age at onset were evaluated. Detailed information about the genetic study was provided to all participants, who signed written consent forms. This study was approved by the Ethics Committee of Seoul National University Hospital. Subjects with a history of any kind of organic abnormality of the brain, alcohol-related mental problems, drug abuse, or other physical illnesses possibly manifesting with psychiatric symptoms were not included in this study. Finally, 180 patients with schizophrenia (113 males, 67 females) were included. The mean age of the male patients was 33.50±7.06 years and that of females was 35.55±7.88 years (p=0.07). The mean age at onset of schizophrenia was 21.88±4.97 years for males and 24.22±6.18 years for females. A significant difference in age at onset was found between male and female patients (p=0.02).

### Obstetric complications

The information on obstetric complications associated with each patient's birth was collected from the mother through direct or phone interviews. We used the Lewis scale^23^ to collect prenatal, perinatal, and postnatal information on the patients. The Lewis scale consists of 16 individual items including information about rubella, syphilis, Rh incompatibility, pregnancy-induced hypertension, obstetric bleeding, premature membrane rupture, labor duration, twin birth, cord prolapse, gestational age, Cesarean section, breech or abnormal presentation, instrumental delivery, birth weight, fetal distress, and gross physical anomaly. Each item was evaluated as definite (score 2), equivocal (score 1), or absent (score 0). The total Lewis score was obtained by summing scores on the individual items.

### Genotyping

DNA was extracted from blood samples using a DNA isolation kit (Roche, Mannheim, Germany). We selected six SNPs for the AKT1 gene: SNP1 (rs3803300), SNP2 (rs1130214), SNP3 (rs3730358), SNP4 (rs1130233), SNP5 (rs2494732), and SNPA (rs2498804). We selected those SNPs because SNP 1-5 have been studied in most of the papers on AKT and schizophrenia. The SNP A is included because it was studied in a Japanese association study of schizophrenia.^19^ SNP1 and SNP4 were the SNPs found to be associated with serious obstetric complications in Nicodemus' paper.^7^ They are located in the 5'-UTR, intron 1, intron 3, exon 9, intron 11, and 3'-UTR, respectively. TaqMan SNP genotyping assays were performed according to the protocol of Applied Biosystems (Foster City, CA, USA). Table 1 shows the location and alleles of the six SNPs selected for this study.

### Statistical analysis

The Hardy-Weinberg equilibrium was tested to detect any possible genetic deviation in the study sample. The comparison of allele and genotype frequencies between patients with a high Lewis score (≥3) and patients with a low Lewis score (0-2) was performed by the chi-square test. If the cell counts less than 5, the significance from Fisher's exact test was adopted. In this study, patients with a high Lewis score were those with obstetric complications. The association between genotype and Lewis score was also analyzed by an analysis of variance (ANOVA). In this case, the obstetric complication (Lewis score) was treated as a continuous variable. Because a positive association was found only for the female patients, the linkage disequilibrium (LD) calculation between SNPs and the haplotype association test was done for female patients using UNPHASED 3.0.13 (http://www.mrc-bsu.cam.ac.uk/personal/frank/software/unphased/).^23^ The haplotype association was tested for two to six SNPs with sliding window methods. In this study the program calculated chi-square and p-value for a specific haplotype association with phenotype in the model of haplotype main effect. Usually LD was measured in both D' and r^2^. D' means a measure of the proportion of the maximum possible LD given the allele frequencies^24^ and r^2^ indicates a squared correlation coefficient between two loci. UNPHASED program analysed both D' and r^2^ between two loci.

## Results

The total Lewis scores ranged from 0 to 8. No difference was found between the mean total Lewis scores for males (1.30±1.61) and females (1.54±1.87; p=0.37). About 10% of patients showed relatively high Lewis scores (≥3; 12.39% in male; 13.43% in female). More than one-third of patients had a total Lewis score of 0 (43.36% in male, 38.81% in female), indicating that their mother reported no obstetric complications.

No correlation was found between total Lewis score and age at onset in male patients, whereas a higher total Lewis score tended to be correlated with earlier age at onset in females (Pearson correlation coefficient=-0.21, p=0.06).

No deviations from the Hardy-Weinberg equilibrium were observed for the six SNPs of the AKT1 gene (data not shown). When the allelic distribution was compared between high and low total Lewis score in the total sample, no SNP was associated with obstetric complications. However, an additional analysis of the male and female subgroups found a significant association between SNPA and SNP4 and Lewis score in the female group only (p=0.02 for SNPA, p=0.04 for SNP4; Table 2). No significant association by genotype distribution was observed for any of the SNPs (Table 3). However, an ANOVA between genotype and Lewis score found that SNPA was significantly associated with obstetric complications in the total sample (F=3.64, df=2, p=0.03), and a tendency was found for an association in the female patient group (F=2.73, df=2, p=0.07). This is consistent with the finding of allelic association with SNPA. However, we found no genotypic association for SNP4 in any subgroup of the sample. Because more evidence supported a positive association in females, the haplotype analysis was performed only for the female subgroup. SNP4 and SNP5 were closely adjacent to each other and the SNP5-SNPA haplotype showed a positive association with obstetric complications (p=0.03). The SNP4-SNP5 and SNP4-SNP5-SNPA haplotypes also showed positive associations (p=0.05; Table 4).

## Discussion

We found an association in female patients with schizophrenia between the SNPs in the AKT1 gene and total Lewis score measuring obstetric complications. Considering the allele, genotype, and haplotype association results, SNPA and SNP4 seem likely to be associated with obstetric complications. Although we could not investigate gene-environment interaction because of the lack of data, our finding indirectly implicates a possible role for the AKT1 gene in obstetric complications and schizophrenia. Because these findings did not survive a correction for multiple testing, their meaning should be interpreted carefully, and replication studies are required.

Numerous endogenous stressors may occur during fetal brain development, and dysregulation of neuroprotection could cause an abnormality in brain development that may result in schizophrenia. There is significant supporting evidence, such as ventricular enlargement and decreased hippocampal volume,^26^-^28^ that schizophrenia is caused by hypoxia-ischemia during neural development; hence, it would be reasonable to speculate that neurovascular units would be important to normal brain development. Interestingly many genes play roles in both neurons and in vascular endothelial cells, and neuronal and vascular development have been discovered to be parallel.^29^ AKT1 is one of many candidate genes in schizophrenia that are likely regulated by hypoxia and expressed in vascular systems.^5^

Our study suggested a possible role for the AKT1 gene in obstetric complications; the association was more notable in female patients with schizophrenia. Gender differences in hypoxia-related brain insult have been suggested by previous studies. After neonatal hypoxic-ischemia, apoptotic mechanisms may be activated differently in male and female brains, but the mechanism is not clear.^30^ Differences in genetic association for hypoxic obstetric complications would be a possible cause for this kind of gender difference.

There are several limitations to this study. First, no Lewis scale data were available for the control sample, and we could not include genotype data from control subjects for our analysis. Therefore, an analysis of the gene-environment interaction, i.e. AKT1 SNPs genotype-Lewis score for obstetric complications, could not be performed. Second, the Lewis scale was only available for 180 patients with schizophrenia. This sample size is not large to draw statistically valid conclusions, especially with rare alleles or haplotype effects on obstetric complications. Third, the information on obstetric complications was collected retrospectively from patients' mothers. Even though some studies have found that mothers were able to recall obstetric complications decades after birth,^31^,^32^ these retrospective data would have some bias.

Fetal hypoxia has been considered an important environmental risk factor for schizophrenia. Now it should be considered as an intermediate result of specific gene mutations. The genes involved are likely to be genes with dual neural and vascular-cell function. A dysregulation in AKT1 function, possibly due to genetic mutation, could cause schizophrenia developmentally through a hypoxic insult to the fetal brain or through other genetic effects on the brain function of schizophrenic patients. Many candidate genes may need to be tested to examine this hypothesis. Genetic factors play a role in environmental exposure of the subject has been hypothesized and is fairly widely accepted.^33^ From this perspective, more studies on genes and various environmental stressors are warranted to understand the multiple-layered and complex nature of the gene-environment interaction.

## Figures and Tables

**TABLE 1:**
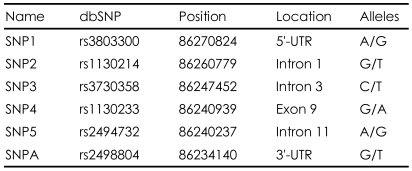
Six single nucleotide polymorphisms (SNPs) of the AKT1 gene

**TABLE 2:**
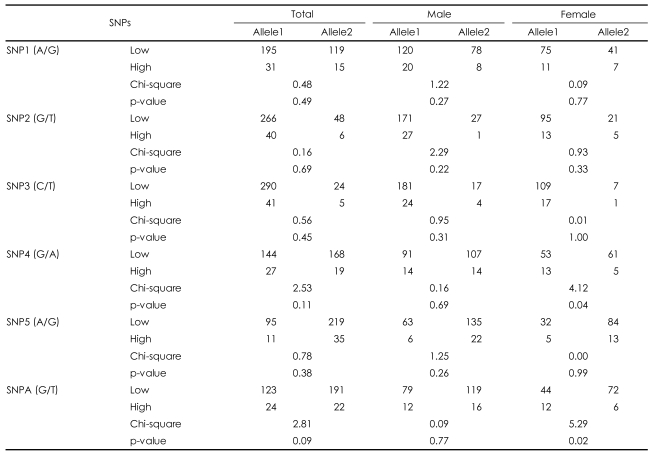
Allele distribution of AKT1 gene single nucleotide polymorphisms (SNPs) with obstetric complications

SNP1: rs3803300, SNP2: rs1130214, SNP3: rs3730358, SNP4: rs1130233, SNP5: rs2494732, SNPA: rs2498804, Low: group of schizophrenic patients with Lewis score (0-2), High: group of schizophrenic patients with Lewis score (≥3)

**TABLE 3:**
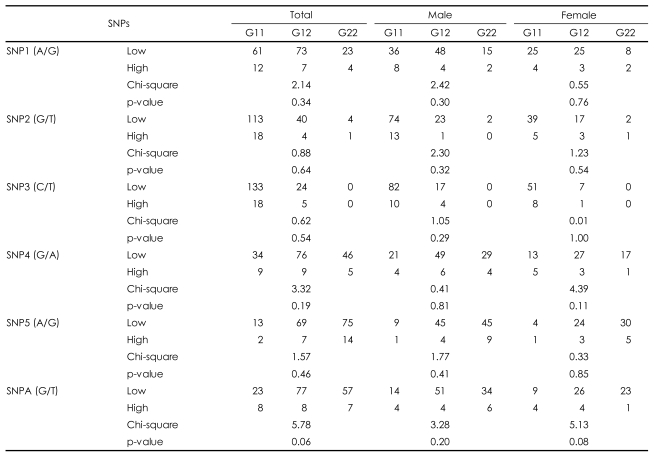
Genotype distribution of the AKT1 gene single nucleotide polymorphisms (SNPs) with obstetric complications

SNP1: rs3803300, SNP2: rs1130214, SNP3: rs3730358, SNP4: rs1130233, SNP5: rs2494732, SNPA: rs2498804, Low: group of schizophrenic patients with Lewis score (0-2), High: group of schizophrenic patients with Lewis score (≥3), G11: genotype with homozygote of allele 1, G12: genotype with heterozygote of allele 1 and allele 2, G22: genotype with homozygote of allele 2

**TABLE 4:**
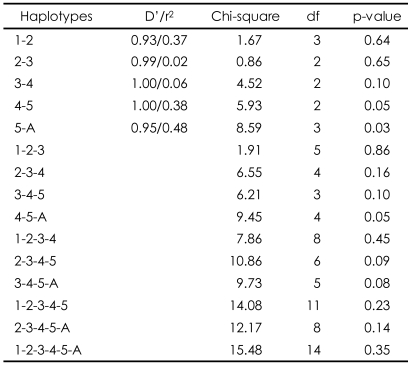
Haplotypic associations in female schizophrenic patients

1: SNP1, 2: SNP2, 3: SNP3, 4: SNP4, 5: SNP5, A: SNPA, D': a measure of the proportion of the maximum possible LD given the allele frequencies, r^2^: a squared correlation coefficient between two loci, SNP: single nucleotide polymorphism
